# A Benchmark for Entropy Estimators

**DOI:** 10.3390/e28030311

**Published:** 2026-03-10

**Authors:** Lucio M. Calcagnile, Angelo Di Garbo, Stefano Galatolo

**Affiliations:** 1 Istituto di Biofisica, CNR, Via G. Moruzzi 1, 56124 Pisa, Italy; 2 Dipartimento di Fisica, Università di Pisa, Via Buonarroti 1, 56127 Pisa, Italy; 3 Dipartimento di Matematica, Università di Pisa, Via Buonarroti 1, 56127 Pisa, Italy

**Keywords:** Kolmogorov–Sinai entropy, Lyapunov exponent, complexity, time series

## Abstract

This study assessed the performance of several entropy estimators for numerical time series and symbolic data on non-trivial one-dimensional dynamical systems whose Kolmogorov–Sinai entropy is known with certified accuracy: recent computer-assisted proof techniques provide rigorous values together with explicit error bounds. We considered four classes of interval maps, including piecewise expanding maps with and without a Markov partition and an intermittent Pomeau–Manneville map, and generated long orbits for each system. We then compared the certified entropy with the output of widely used estimators: Approximate Entropy, Sample Entropy, Permutation Entropy, a symbolic Plug-In estimator of the entropy rate, and the Non-Sequential Recursive Pair Substitution (NSRPS) method (the latter two with Grassberger-type bias correction). Our experiments reveal substantial, dynamics-dependent differences in accuracy and robustness. In particular, Approximate Entropy and the symbolic methods (Plug-In and NSRPS) consistently yielded estimates within the rigorous error bars across all systems, whereas Sample Entropy showed a marked systematic underestimation, and Permutation Entropy exhibited large biases, especially for expanding maps without a Markov partition. The resulting benchmark provides a quantitative testbed for evaluating entropy estimation techniques in deterministic dynamical systems.

## 1. Introduction

In recent years, advances in validated numerics and computer-assisted proofs have made it possible to obtain mathematically rigorous estimates of several key dynamical indicators, including Lyapunov exponents and Kolmogorov–Sinai (KS) entropy, for non-trivial dynamical systems (see, e.g., [1,2,3,4]). This progress relies on a careful combination of a posteriori error estimates, interval arithmetics (see [5]), and suitably designed numerical schemes. Such rigorous computations provide reliable bounds on the quantities of interest and are used here as a solid foundation for benchmarking and validating statistical methods used in time-series analysis.

Estimating entropy from observed data is a classical and still very active topic in nonlinear time-series analysis and information theory. In many applications, from physics and engineering to physiology and neuroscience, the underlying dynamics are unknown or only partially specified, and entropy-like quantities are used as empirical measures of complexity, irregularity, or information production [6,7]. This has motivated the development of a wide range of statistical estimators of entropy and entropy rates, tailored to different types of data (real-valued or symbolic, short or long, noisy or noise-free) and embodied various modelling assumptions.

Among the most popular approaches, Approximate Entropy (ApEn) and Sample Entropy (SampEn) were introduced to quantify the regularity and complexity of finite, typically noisy physiological time series [8,9]. Their definitions are based on the frequency of recurrent patterns in delay-embedded vectors, controlled by embedding dimension and radius parameters, and they have been applied in numerous empirical studies [10,11,12]. Permutation Entropy (PerEn), in contrast, relies on the ordinal structure of local patterns in a time series and provides a simple, fast, and robust complexity measure that is invariant under monotone transformations of the data [13,14]. In another line of work, entropy-rate estimators based on symbolic data and information-theoretic methods have been developed, including Plug-In (or block-frequency) estimators, sometimes enhanced by bias-correction techniques, as well as compression-based methods and substitution-based methods such as the Non-Sequential Recursive Pair Substitution (NSRPS) algorithm. These approaches have been analysed and compared in several works [15,16,17].

While these methods are widely used, their performance can depend strongly on the statistical properties of the underlying dynamical process, on the length and resolution of the data, and on the choice of hyperparameters. For this reason, it is important to have carefully designed benchmarks that allow for a quantitative comparison of different estimators under controlled conditions. In particular, when the data are generated by a deterministic dynamical system for which the true entropy is known (or at least bounded) with rigorous error estimates, one can evaluate not only the relative performance of different estimators but also their absolute accuracy with respect to the mathematically certified ground truth. This is precisely the perspective advocated in [2], where rigorous entropy estimates obtained via transfer-operator techniques were proposed as a benchmark for time-series methods.

The goal of the present study is to contribute to this program by constructing such a benchmark for a set of one-dimensional chaotic maps and by systematically comparing a group of widely used entropy estimators on time series generated by these systems, with the ground truth provided by the rigorous estimates available on those systems.

More specifically, we considered three qualitatively different classes of interval maps: (i) piecewise expanding maps admitting a Markov partition, (ii) piecewise expanding maps without the Markov property, and (iii) intermittent maps of Pomeau–Manneville type, displaying a neutral fixed point and slow (typically polynomial) decay of correlations. For each of these systems, we relied on existing computer-assisted results to obtain a certified value of the KS entropy, either directly or via rigorous computation of the invariant density and of the Lyapunov exponent [2]. These certified values provided the reference against which we compared the output of the statistical estimators.

On the data-analysis side, we worked both with numerical (real-valued) time series and with symbolic sequences obtained from suitable partitions. On these data we applied the estimators mentioned above: ApEn and SampEn, as originally defined in [8,9]; PerEn in the sense of Bandt and Pompe [13]; a symbolic Plug-In estimator of the entropy rate based on empirical block frequencies, with finite-sample bias corrections, inspired by information-theoretic approaches [16]; and the Non-Sequential Recursive Pair Substitution (NSRPS) method, which estimates entropy via a recursive symbol-substitution scheme [15], again with finite-sample bias corrections. This selection allowed us to compare methods tailored to real valued time series with more traditional symbolic estimators, as well as to contrast “local” pattern-based approaches (ApEn, SampEn, PerEn) with global, compression-like methods (Plug-In and NSRPS).

Our numerical experiments showed that the behaviour of these estimators can vary significantly across different systems and regimes. Even when all methods were provided with long, noise-free trajectories, the resulting entropy estimates exhibited non-negligible biases and different degrees of robustness with respect to parameter choices. In particular, we found that the ApEn and the symbolic estimators (Plug-In, NSRPS) tended to track the certified entropy values more closely and more consistently across the examples considered, while PerEn and SampEn could display systematic underestimation. In particular PerEn can be affected by large biases when applied to expanding maps without a Markov partition.

Beyond the empirical estimators analysed in this paper, several alternative approaches are commonly used to estimate the entropy of a time series. These methods include (i) Markov and variable-memory models, where the entropy rate is computed from estimated transition probabilities [18,19,20]; (ii) hidden Markov models and related latent-state models, for which entropy rates can be studied/approximated via filtering or matrix-product techniques [21,22]; (iii) Lempel–Ziv/match-length estimators (compression-based), which are consistent for broad classes of stationary ergodic sources [23,24,25,26]; (iv) *k*-nearest-neighbour estimators for (differential) entropy and related information quantities, often used after delay embedding [27,28]; and (v) Bayesian entropy estimators for undersampled discrete distributions (e.g., NSB-type hierarchical priors) [29,30]. We hope that this work will stimulate further research on systematic, reproducible benchmarking of entropy-rate estimators beyond the methods considered here. Regarding Markov models, we further note that the certified entropy bounds in [2] rely on an Ulam-type discretization [31], whereby the Perron–Frobenius (transfer) operator of the dynamical system is approximated by a finite-dimensional stochastic matrix, i.e., by a finite-state Markov chain. This construction however is not a time-series estimator: it operates at the operator level (a Galerkin/finite-dimensional reduction of the transfer operator) rather than on a single observed trajectory. When combined with explicit, rigorous bounds on the discretization error, this approach yields certified approximation intervals for dynamical invariants such as the entropy for suitable classes of systems; we refer to [2] for details.

The structure of the paper is as follows. In Section 2 we recall the main entropy-related quantities used in this work, together with their mathematical definitions, and introduce the time-series estimators that will be compared: ApEn, SampEn, PerEn, and the symbolic estimators (Plug-In and NSRPS). Section 3 describes the dynamical systems employed to generate the data, namely a piecewise expanding map with a Markov partition, an expanding map without the Markov property, a nonlinear variant of the beta map, and a Pomeau–Manneville map. For each system we briefly recall the available rigorous results on the invariant measure and entropy. Section 4 presents the numerical experiments, detailing the data generation, the choice of parameters for each estimator, and the comparison between the resulting estimates and the certified entropy values. Finally, in Section 5 we summarise our findings, discuss their implications for the practical use of entropy estimators in deterministic dynamics, and outline several directions for further work. Lastly, in Appendix A, Appendix B and Appendix C more details can be found concerning the linear autocorrelation properties of the time series (Appendix A); the dependence of ApEn, SampEn, and PerEn on the parameter values (Appendix B); and how the parameters of the symbolic methods were chosen (Appendix C).

We emphasise that the present study was deliberately restricted to a specific, albeit diverse, class of one-dimensional dynamical systems for which certified estimates on the entropy were available in the literature. The examples considered range from uniformly expanding maps with exponential decay of correlations to intermittent systems with polynomial mixing rates and thus already exhibit markedly different statistical behaviours. Nevertheless, given this restricted scope, our results should not be interpreted as providing a definitive, global ranking of the performance of the various estimators. Rather, they should be viewed as a first, quantitatively controlled step towards such an assessment. The strength of our benchmark lies in the fact that the “true” entropy values used for comparison were obtained via rigorous computation, with fully certified error bounds. Extending this approach to higher-dimensional systems, to data contaminated by noise, and to other classes of observables will require further work, but we believe that the methodology developed here provides a solid starting point for such future investigations.

## 2. General Concepts and Entropy Estimators

As discussed, in this study several well-known methods of time series analysis were employed to quantify the complexity of signals generated by specific dynamical systems with the aim of comparing their performance. Therefore it is useful to introduce the entropy estimators used and compared in the paper and outline some general technical issues arising.

### 2.1. Kolmogorov–Sinai Entropy

Let us introduce the general mathematical framework that is at the base of the contents of this work. Let (X,B,μ,T) be a dynamical system where *X* is the set of states; B is the sigma algebra of all measurable subsets of *X*, μ is a probability measure defined on B, and T:X⟶X represents the deterministic rule defining the time evolution of the states. The measure μ is said to be *T*-invariant (or, equivalently, the map *T* is said to be measure-preserving) if

$$\mu (T^{-1}\left(B\right))=\mu \left(B\right)\;\mathrm{for}\;\mathrm{every}\;B\in B,$$

where $T^{-1}\left(B\right)=\left\{x\in X:T\left(x\right)\in B\right\}$ is the preimage of *B* under *T*. For each finite partition $P=\{P_{1},P_{2},\ldots ,P_{L}\}\subset B$, the corresponding Shannon entropy is defined as

(1) $$\begin{array}{c} H\left(P\right)=-\sum_{j=1}^{L}\mu \left(P_{j}\right)\log\left(\mu \left(P_{j}\right)\right) \end{array}$$

with the prescription that 0log0=0. Now, using this partition, the complexity of the dynamics generated by the map *T* can be quantified as follows. Let A={1,2,…,L} be an alphabet; then to each state $x\in P_{j}\;\left(j=1,2,\ldots ,L\right)$ is associated with the symbol j∈A. Then the idea is to generate a new partition of *X* containing information on the time evolution of the state determined by the map *T*. To this aim the collection of sets

(2) $$\begin{array}{c} P_{a_{1},a_{2},\ldots ,a_{n}}=\left\{x\in X|x\in P_{a_{1}},T\left(x\right)\in P_{a_{2}},\ldots ,T^{n-1}\left(x\right)\in P_{a_{n}}\right\} \end{array}$$

defines a finer partition of *X*, where $a_{j}\in A$ and $T^{k}$ stand for the *k*-times composition of the map [32]. With the above definitions, the complexity of the dynamics generated by the map *T* is defined as follows:

(3) $$\begin{array}{c} H\left(T,P\right)=\lim_{n\to +\infty }\frac{H(T,P_{Q_{n}})}{n} \end{array}$$

where $Q_{n}=\left(a_{1},a_{2},\ldots ,a_{n}\right)$ defines a given combination of indices and $H\left(T,P_{Q_{n}}\right)=-\sum_{Q_{n}}\mu \left(P_{Q_{n}}\right)\log\mu \left(P_{Q_{n}}\right)$. It can be proven that the above limit exists [32]. Since the value of H(T,P) depends on the choice of the original partition $P=\{P_{1},P_{2},\ldots ,P_{L}\}\subset B$, the Kolmogorov–Sinai entropy is defined as

(4) $$\begin{array}{c} H\left(T\right)=\underset{P\in \bar{P}}{\sup}H\left(T,P\right) \end{array}$$

where $\bar{P}$ is the set of all finite measurable partitions of *X*. The calculation of the Kolmogorov–Sinai entropy from Equation (4) is in general very difficult. However, if a generating partition *G* of *X* is known, then the equality H(T)=H(T,G) holds [32]. We conclude by remarking that the intrinsic difficulties to calculate H(T) starting from Equation (4) prompted the search for alternative and more direct methods for estimating the KS entropy.

### 2.2. Approximate Entropy and Sample Entropy

In the spirit of the last remark of the previous section, in the following we describe the ApEn and SampEn complexity measures [8,9]. It is useful, before defining explicitly how the above quantity can be estimated from a time series, to state some general definitions and results. As shown in [8,9], the ApEn is an approximation of the Eckmann–Ruelle entropy [33], while the SampEn is an approximation of the $H_{2}$ entropy [34,35,36,37]. Let (X,B,μ,T) be a dynamical system and assume that *X* is equipped with the metric $\sigma :X\times X⟶R^{+}\cup \left\{0\right\}$. Using this metric, we define a new metric depending on the dynamical properties of the map T:X⟶X. More precisely, for any integer k≥1, let us define this last metric as

(5) $$\begin{array}{c} \sigma_{k}\left(x,x^{\prime }\right)=\max_{0\leq i\leq k-1}\sigma \left(T^{i}\left(x\right),T^{i}\left(x^{\prime }\right)\right) \end{array}$$

where $T^{i}$ stands for the composition of the map *i* times. Moreover, for each x∈X and r>0, let us define the following set

(6) $$\begin{array}{c} B_{k}\left(x,r\right)=\left\{x^{\prime }\in X\;|\;\sigma_{k}\left(x,x^{\prime }\right)<r\right\} \end{array}$$

representing the open ball centred in *x*. If *X* is a compact metric space with metric σ, the correlation entropy ($E_{C}$) and the $H_{2}$ entropy [34,36] are defined as

(7) $$\begin{array}{c} E_{C}=\underset{r\to 0}{lim sup}\underset{k\to +\infty }{lim sup}-\frac{1}{k}\int_{X}\ln\left(\mu \left(B_{k}\left(x,r\right)\right)\right)d\mu \left(x\right) \end{array}$$

(8) $$\begin{array}{c} H_{2}=\underset{r\to 0}{lim sup}\underset{k\to +\infty }{lim sup}-\frac{1}{k}\ln\int_{X}\mu \left(B_{k}\left(x,r\right)\right)d\mu \left(x\right). \end{array}$$

In general it holds that $H_{2}\leq E_{C}\leq H\left(T\right)$ [34,36]. However, if μ is invariant and nonatomic, the Brin–Katok theorem [38] implies that $E_{C}=H\left(T\right)$ [36]. Starting from the analytical expression defining the correlation entropy and using the equality $E_{C}=H\left(T\right)$, we can proved that for an ergodic map *T*, the Kolmogorov–Sinai entropy can be expressed as

(9) $$\begin{array}{c} H\left(T\right)=\lim_{r\to 0}\lim_{k\to +\infty }\lim_{n\to +\infty }\frac{1}{n}\left(\sum_{j=0}^{n-1}\ln\mu \left(B_{k}\left(T^{j}\left(x\right),r\right)\right)\right.\\ -\left.\sum_{j=0}^{n-1}\ln\mu \left(B_{k+1}\left(T^{j}\left(x\right),r\right)\right)\right). \end{array}$$

To prove the above results, it is assumed that the following limit exists.

(10) $$\begin{array}{c} \lim_{k\to +\infty }\lim_{n\to +\infty }\frac{1}{n}\left(\sum_{j=0}^{n-1}\ln\mu \left(B_{k}\left(T^{j}\left(x\right),r\right)\right)-\sum_{j=0}^{n-1}\ln\mu \left(B_{k+1}\left(T^{j}\left(x\right),r\right)\right)\right) \end{array}$$

Let us now show how to connect the above measures of complexity with the problem of quantifying the complexity of time series. Let ϕ:X⟶R be an observable of a dynamical system and $x_{i},\;i=1,2,\ldots ,N$ be a time series obtained by sampling the values of ϕ at a fixed time interval Δt. Moreover, assume that the dynamics is confined in a compact attractor *A*. Then, the Takens theorem ensures us that the set of all embedding vectors $v_{i}\left(E\right)=\left(x_{i},x_{i+1},x_{i+2},\ldots ,x_{i+E-1}\right)\in R^{E},\;i=1,2,\ldots$, (i.e., the reconstructed phase space) is diffeomorphic to the original attractor A if $E>2d_{A}$, where $d_{A}$ is the dimension of *A* [34,35,36]. Therefore, to quantify the complexity of the time series $x_{i},\;i=1,2,\ldots ,N$, we can use Equation (10) in the reconstructed phase space with the metric defined by $\bar{\sigma }\left(v_{i}\left(E\right),v_{j}\left(E\right)\right)=\max_{k=0,1,\ldots ,E-1}\left|x_{i+k}-x_{j+k}\right|$. Consequently, for an ergodic *T* we can calculate the complexity of the dynamics generated by the map through the computation of the Kolmogorov–Sinai entropy using the time series $x_{i},\;i=1,2,\ldots ,N$. But, in order to apply (10), we need a way to calculate $\mu \left(B_{k}\left(T^{j}\left(x\right),r\right)\right)$. For an ergodic map *T*, the value of such a quantity can be estimated by using the following expression

(11) $$\begin{array}{c} \;\;C\left(j,E,r,{\left\{x_{l}\right\}}_{l=1}^{N}\right)=\frac{\#\left\{1\leq k\leq N-E+1:\bar{\sigma }\left(v_{j}\left(E\right),v_{k}\left(E\right)\right)\leq r\right\}}{N-E+1} \end{array}$$

where the symbol # indicates the set cardinality. Then substituting in Equation (10), we get an approximation of the Kolmogorov–Sinai entropy for a given radius *r*. As proposed in [8], the evaluation of the above expression for finite values of *E* and *N* and for a given $r\in R^{+}$ leads to the so called ApEn complexity measure for a time series defined as

(12) $$\begin{array}{c} \mathrm{ApEn}\left(E,r,{\left\{x_{l}\right\}}_{l=1}^{N}\right)=\frac{\sum_{i=1}^{N-E+1}\ln C\left(i,E,r,{\left\{x_{l}\right\}}_{l=1}^{N}\right)}{N-E+1}\\ -\frac{\sum_{i=1}^{N-E}\ln C\left(i,E+1,r,{\left\{x_{l}\right\}}_{l=1}^{N}\right)}{N-E}. \end{array}$$

Similarly, as shown in [9,34,35,37], the $H_{2}$ entropy of a time series can be estimated as

(13) $$\begin{array}{c} H_{2}=\lim_{N\to +\infty }\ln\frac{\bar{C}\left(E,r,{\left\{x_{l}\right\}}_{l=1}^{N}\right)}{\bar{C}\left(E+1,r,{\left\{x_{l}\right\}}_{l=1}^{N}\right)} \end{array}$$

where $\bar{C}\left(E,r,{\left\{x_{l}\right\}}_{l=1}^{N}\right)$ is given by

(14) $$\begin{array}{c} \bar{C}\left(E,r,{\left\{x_{l}\right\}}_{l=1}^{N}\right)=\\ \frac{2\#\left\{\left(i,j\right):1\leq i<j\leq N-E+1:\bar{\sigma }\left(v_{i}\left(E\right),v_{j}\left(E\right)\right)\leq r\right\}}{N-E+1}. \end{array}$$

Then, as proposed in [9], the Sample Entropy of a time series for given values of N,E,r is defined as follows

(15) $$\begin{array}{c} \mathrm{SampEn}\left(E,r,{\left\{x_{l}\right\}}_{l=1}^{N}\right)=\ln\bar{C}\left(E,r,{\left\{x_{l}\right\}}_{l=1}^{N}\right)-\ln\bar{C}\left(E+1,r,{\left\{x_{l}\right\}}_{l=1}^{N}\right). \end{array}$$

According to [8,9], when these two measures of entropy are applied to time series, then it is suggested to adopt the values of the parameters, *E* and *r*: E=2 and r∈[0.1σ,0.25σ], where σ is the standard deviation of the time series.

### 2.3. Permutation Entropy

The Permutation Entropy (PerEn) was introduced in [13] as a measure of complexity of time series and can be used to estimate the Kolmogorov–Sinai entropy [14,39]. Let ${\left\{x_{l}\right\}}_{l=1}^{N}$ be a time series and $v_{i}\left(E,\tau \right)=\left(x_{i},x_{i+\tau },x_{i+2\tau },\ldots ,x_{i+(E-1)\tau }\right)\in R^{E}$ the i−th reconstructed state, where τ=1,2,… is the lag time, *E* is the embedding dimension, and 1≤i≤N−(E−1)τ. Let $A=\left\{1,2,\ldots ,E\right\}$ and $\Pi_{E}$ be the set of all permutations of the elements of *A*. Let us define a map that to each $v_{i}\left(E,\tau \right)$ associates an element of $\Pi_{E}$. This map is defined by employing the ordinal ordering pattern of the components of the vector $v_{i}\left(E,\tau \right)$. More precisely, let $r_{1}^{i}\in A$ denote the position of the largest of the components of $v_{i}\left(E,\tau \right)$. For instance, if $v_{i}\left(3,1\right)=\left(2,3,1\right)$ then $r_{1}^{i}=2$. Similarly, let $r_{2}^{i}$ be the position of the second largest of the components of $v_{i}\left(E,\tau \right)$. In the previous example it is $r_{2}^{i}=1$. Continuing in this way we get the sequence $\pi_{i}=\left(r_{1}^{i},r_{2}^{i},\ldots ,r_{E}^{i}\right)\in \Pi_{E}$. We have implicitly assumed that all the values of the time series ${\left\{x_{l}\right\}}_{l=1}^{N}$ are different. If such a hypothesis does not hold for two (or more) components, then the ordinal ordering is chosen in this way: if the amplitudes of the components in the positions $r_{n}^{i}$ and $r_{m}^{i}$ are the same, then $r_{m}^{i}=r_{n}^{i}+1$ ($r_{n}^{i}=r_{m}^{i}+1$) if $r_{n}^{i}<r_{m}^{i}$ (if $r_{m}^{i}<r_{n}^{i}$). Using this mapping from the space of all reconstructed states to $\Pi_{E}$, we can estimate the probability of occurrence of a given permutation $\left(r_{1},r_{2},\ldots ,r_{E}\right)\in \Pi_{E}$ (henceforth called a “word”) as

(16) $$\begin{array}{c} p_{k}=\frac{\#\left\{(r_{1},r_{2},\ldots ,r_{E})\right\}}{N-(E-1)\tau } \end{array}$$

where the numerator represents the total number of words $\left(r_{1},r_{2},\ldots ,r_{E}\right)$, and N−(E−1)τ is the total number of words occurring in the given time series. For instance let ${\left\{x_{l}\right\}}_{l=1}^{N}=\left(2,3,1,7,1,5\right)$ be a time series with N=6. Let us consider the case E=4 and τ=1. In this case the total number of reconstructed states is given by N−(E−1)τ=6−(4−1)=3, and they are $v_{1}\left(4,1\right)=\left(2,3,1,7\right)$, $v_{2}\left(4,1\right)=\left(3,1,7,1\right)$, and $v_{3}\left(4,1\right)=\left(1,7,1,5\right)$. Using the previous rules we have the following words: $v_{1}\left(4,1\right)=\left(2,3,1,7\right)⟶\left(4,2,1,3\right)\in \Pi_{4}$, $v_{2}\left(4,1\right)=\left(3,1,7,1\right)⟶\left(3,1,2,4\right)\in \Pi_{4}$. and $v_{3}\left(4,1\right)=\left(1,7,1,5\right)⟶\left(2,4,1,3\right)\in \Pi_{4}$. Using Equation (16) the PerEn of a time series is defined as

(17) $$\begin{array}{c} \mathrm{PerEn}\left(E,\tau ,{\left\{x_{l}\right\}}_{l=1}^{N}\right)=-\frac{1}{E}\sum_{\Pi_{E}}p_{k}\ln p_{k} \end{array}$$

with the prescription 0ln0=0 [13]. For the above example the probability of occurrence of each word is 1/3 and the corresponding value of the Permutation Entropy is $\mathrm{PE}=-0.25\left(\frac{1}{3}\ln\frac{1}{3}+\frac{1}{3}\ln\frac{1}{3}+\frac{1}{3}\ln\frac{1}{3}\right)≃0.27465$. It is worth mentioning that for a measure preserving map *T* of the interval piecewise monotone and continuous, the $\lim_{N\to \infty }\mathrm{PerEn}\left(E,\tau ,{\left\{x_{l}\right\}}_{l=1}^{N}\right)$ is equal to the corresponding value of the Kolmogorov–Sinai entropy [14].

### 2.4. Symbolic Methods

As seen in Section 2.1 a common procedure for estimating the Kolmogorov–Sinai entropy of a dynamical system (X,B,μ,T) from sample orbits involves the discretisation of the phase space and the symbolisation of the orbits. More precisely, let ${\left\{P_{i}\right\}}_{i=1,2,\ldots ,L}$ be a finite partition of *X* with the properties

$$X=\overset{L}{\underset{i=1}{⋃}}P_{i},\;P_{i}\cap P_{j}=\emptyset \;\mathrm{if}\;i\neq j,$$

and A={1,2,…,L} be the alphabet. Then the corresponding symbolic dynamics of (X,B,μ,T) can be defined through the map ϕ(x)=j if $x\in P_{j}$. This way, an orbit $x={\left(T^{n}\left(x\right)\right)}_{n\in N}$ of the dynamical system can be encoded into a symbolic sequence $s={\left(j_{n}\right)}_{n\in N}$, with $j_{n}\in A$,

$$x=\left(x,T\left(x\right),T^{2}\left(x\right),\ldots \right)\overset{S_{T}}{\to }s=\left(j_{0},j_{1},j_{2},\ldots \right)\;\mathrm{with}\;T^{n}\left(x\right)\in P_{j_{n}},$$

thus transferring the dynamical properties of the original system into symbolic dynamics. The derived symbolic system can be regarded as a finite-alphabet information source, that is, as a stationary process ${\left(S_{n}\right)}_{n\in N}$ over a finite alphabet *A*. Indicating with $\Sigma =A^{N}$ the set of all infinite sequences of elements of *A*, with C the σ-algebra generated by the cylinder sets $\{S_{n}=j\}\subset \Sigma$ (n∈N,j∈A), with ν the measure defined by $\nu \left(C\right)=\mu (S_{T}^{-1}\left(C\right))$, and with σ:Σ→Σ the (left) shift operator defined by ${\left(\sigma s\right)}_{n}={\left(s\right)}_{n+1}$, the dynamical system (X,B,μ,T) translates into the symbolic dynamical system (Σ,C,ν,σ).

Since the derived symbolic dynamical system determines a stationary process ${\left(S_{n}\right)}_{n\in N}$, its block entropy and entropy rate are well defined. As long as the chosen partition $\left\{P_{1},P_{2},\ldots ,P_{L}\right\}$ is generating with respect to B, the entropy rate of the symbolic system equals the Kolmogorov–Sinai entropy of the dynamical system [32]. Based on this equality, one can rely on the entropy estimation of the derived symbolic system, which we carry out with the methods described in Section 2.4.1 and Section 2.4.2.

Recall that given a stationary process $S={\left(S_{n}\right)}_{n\in N}$ on a finite alphabet, its block entropy of order *k* is defined as the Shannon entropy

(18) $$H_{k}\left(S\right)=H\left(S_{n},\ldots ,S_{n+k-1}\right)$$

Moreover, its conditional entropy of order *k* is defined as

(19) $$h_{k}\left(S\right)=H_{k+1}\left(S\right)-H_{k}\left(S\right).$$

It can be shown (see [40]) that as the block length *k* goes to infinity, both $\frac{H_{k}\left(S\right)}{k}$ and $h_{k}\left(S\right)$ converge to the same limit, which is called the *entropy rate* of the process:

(20) $$h\left(S\right)=\lim_{k\to \infty }\frac{H_{k}\left(S\right)}{k}=\lim_{k\to \infty }h_{k}\left(S\right).$$

#### 2.4.1. Plug-In Estimator

In practical applications the entropy rate is approximated taking the values $\frac{H_{k}}{k}$ or $H_{k+1}-H_{k}$ for some *k*. In order for them to be good estimates of the entropy rate, it is necessary that the chosen order *k* be large enough to account for the dependency properties of the process. It can be taken as low as 1 for an i.i.d. process, while it should be much larger for long-memory processes.

On the other hand, one should have sufficient statistics to accurately estimate the probabilities of the *k*-blocks so that *k* should be sufficiently small with respect to the length of the sample *N*.

If N→∞ and k(N)→∞ with $k\left(N\right)\leq \frac{\log N}{h}$, then the entropy estimates $\frac{H_{k(N)}}{k(N)}$ and $h_{k(N)}$ of sample frequencies converge to the entropy rate almost perfectly [40].

Having a finite sample realisation of the process, a Plug-In estimator of the entropy $H_{k}$ is the entropy of the distribution of the *k*-blocks whose probabilities coincide with the relative frequencies observed in the sample.

We rely on Equation (20) for our estimates with the Plug-In estimator, i.e., we take as our best estimate of the entropy rate the conditional entropy $h_{k}$ for some *k*. In Appendix C we detail on how we choose such order *k*.

Since the naive Plug-In estimator systematically underestimates the Shannon entropy of finite samples, in order to reduce the bias, we use the Grassberger correction (see the estimator ${\hat{H}}_{G}$ in [41]).

#### 2.4.2. Non-Sequential Recursive Pair Substitution

Given a symbolic time series, Non-Sequential Recursive Pair Substitution (NSRPS) consists in the iterated procedure of substituting a given character pair (normally the most frequent one) with a new symbol.

For example, starting from the binary string

$$011010111011000111011010011\in A_{0}^{*}={\left\{0,1\right\}}^{*},$$

performing the substitution 01↦2 leads to

$$2122112100211212021\in A_{1}^{*}={\left\{0,1,2\right\}}^{*}.$$

A second substitution 21↦3 would lead to

$$3231300313203\in A_{2}^{*}={\left\{0,1,2,3\right\}}^{*}.$$

Continuing this way, the significant dependency information contained in the time series is condensed into shorter and shorter sequences. In fact, each pair substitution concentrates the information, and this suggests that a sequence of pair substitutions might asymptotically transfer *all* the information into the distribution of pairs.

In [15] it was shown how NSRPS can yield efficient data compression and entropy estimates, and it was conjectured that, under the NSRPS procedure, a process becomes asymptotically Markov. Similarly, in [42] the NSRPS method was studied in the formal context of probability theory, and it was proven that an ergodic process does not go asymptotically Markov as the number of pair substitution steps goes to infinity.

A bit more formally, if at each step *k* the substitution of a pair with maximum probability among all the pairs is performed, then

(21) $$h\left(\nu \right)=\lim_{k\to \infty }\frac{h_{1}\left(\nu_{k}\right)}{{\bar{Z}}_{k}},$$

where ν is the source measure on $A_{0}^{N}$, $\nu_{k}$ is the push-forward measure on $A_{k}^{N}$ induced by the *k*-th pair substitution, $h_{1}=H_{2}-H_{1}$ is the conditional entropy of order 1, and the quantity ${\bar{Z}}_{k}$ is a normalisation factor equal to the inverse of the average shortening [42].

In [17] the NSRPS method was used to estimate the Kolmogorov–Sinai entropy of some maps of the interval, showing that it is in general more efficient and precise than other symbolic methods.

In order to reduce the bias in the calculation of the Shannon entropies in (21) on our finite samples, we again use the Grassberger correction as in the Plug-In method.

## 3. Dynamical Systems Employed to Generate the Time Series

In this paper we apply the entropy estimators described in the previous sections to time series generated by well-defined one-dimensional discrete-time dynamical systems. All analysed time series are univariate and are produced by iterating maps T:[0,1]→[0,1] belonging to three main classes: piecewise expanding maps with a Markov partition, piecewise expanding maps without the Markov property, and an intermittent Pomeau–Manneville map.

For the first three dynamical systems, *T* is a piecewise expanding map of the interval. Roughly speaking, T:[0,1]→[0,1] is piecewise expanding if there exists a finite partition of [0,1] into *L* pairwise disjoint open intervals

$$U_{j}=\left(a_{j},a_{j+1}\right),\;j=1,2,\ldots ,L,$$

with $0=a_{1}<a_{2}<\ldots <a_{L+1}=1$ and $\left[0,1\right]={⋃}_{j=1}^{L}\bar{U_{j}}$, where $\bar{U_{j}}$ is the closure of $U_{j}$, such that the following holds:

- $T_{|U_{j}}$ is continuous and $C^{2}$ for each j=1,…,L.
- There exists λ>1 such that $|T^{\prime }\left(x\right)|\geq \lambda$ for all *x* where $T^{\prime }$ is defined.

These hypotheses guarantee the existence of at least one invariant probability measure μ that is absolutely continuous with respect to the Lebesgue measure and whose density has bounded variation [43]. For the maps adopted here, this invariant measure is unique.

The asymptotic dynamical regime of each of the four one-dimensional maps considered in this work will be characterised by its Lyapunov exponent. For x∈[0,1], the (pointwise) Lyapunov exponent is defined by

$$L_{\exp}\left(x\right)=\lim_{n\to +\infty }\frac{1}{n}\sum_{i=0}^{n-1}\log\left|{\left(T^{i}\right)}^{\prime }\left(x\right)\right|,$$

whenever this limit exists. By the Birkhoff ergodic theorem (see, e.g., [32]), if μ is an ergodic *T*-invariant probability measure and $\log|T^{\prime }|\in L^{1}\left(\mu \right)$, then $L_{\exp}\left(x\right)$ exists for μ-almost every *x* and is μ-almost surely constant:

(22) $$L_{\exp}\left(x\right)=\int_{0}^{1}\log\left|T^{\prime }\left(y\right)\right|\;d\mu \left(y\right)=:L_{\exp},\;\mathrm{for}\;\mu \text{-}a.e.\;x.$$

In particular, the single number $L_{\exp}$ provides a quantitative description of the average exponential separation rate of nearby trajectories with respect to the invariant measure μ.

For the class of interval maps we are interested in, there is a strong relationship between the Lyapunov exponent and the Kolmogorov–Sinai entropy. The following result, Theorem 1, can be seen as a one-dimensional version of Pesin’s entropy formula.

Before stating it, we recall that the Hausdorff dimension of a measure μ, denoted by HD(μ), is defined as the infimum of all dimensions *d* for which the Hausdorff *d*-dimensional measure of the support of μ equals 0 or, equivalently, as the supremum of all dimensions *d* for which the Hausdorff *d*-dimensional measure of the support of μ equals *∞*:

(23) $$\begin{array}{cc} HD(\mu ) & =\inf\{d\geq 0\;|\;H^{d}\left(\mathrm{Supp}\left(\mu \right)\right)=0\}\\ & =\sup\{d\geq 0\;|\;H^{d}\left(\mathrm{Supp}\left(\mu \right)\right)=\infty \}. \end{array}$$

It represents the real value at which the *d*-dimension of the support of μ jumps from 0 (for d<HD(μ)) to *∞* (for d>HD(μ)). It is straightforward to see that, for an absolutely continuous invariant measure μ on the interval, HD(μ)=1.

**Theorem** **1** (Hofbauer–Raith [44])**.** *Let T be a map on [0,1] with finitely many monotonic pieces and a derivative of bounded p-variation for some p>0. If μ is an ergodic T-invariant probability measure with Lyapunov exponent $\lambda_{\mu }>0$, then the Hausdorff dimension of μ satisfies*

$$HD\left(\mu \right)=\frac{h_{\mu }\left(T\right)}{\lambda_{\mu }},$$

*where $h_{\mu }\left(T\right)$ denotes the Kolmogorov–Sinai entropy of (T,μ).*

In particular, for all the maps considered in the following, the invariant measure μ is absolutely continuous with respect to the Lebesgue measure and has a density of bounded variation so that HD(μ)=1. In this case the theorem implies

$$h_{\mu }\left(T\right)=\lambda_{\mu },$$

i.e., the Kolmogorov–Sinai entropy coincides with the Lyapunov exponent. For the Pomeau–Manneville map considered in Section 3.4, a positive Lyapunov exponent and an absolutely continuous invariant measure are also known to exist; again, the entropy can be expressed in terms of the Lyapunov exponent (see, e.g., [45,46] for related one-dimensional Pesin-type formulas). In all four examples, therefore, rigorous bounds on the Lyapunov exponent immediately yield rigorous bounds on the KS entropy, which we use as reference values in the benchmark of Section 4.

Let us now describe the maps of the interval that were employed to generate the time series analysed in the present work.

### 3.1. Lanford Map

The first dynamical system is the Lanford map T:[0,1]→[0,1] [47]. This map is defined by

$$T:x\mapsto 2x+\frac{1}{2}x\left(1-x\right)\;\left(\mathrm{mod}\;1\right).$$

**Figure 1:**
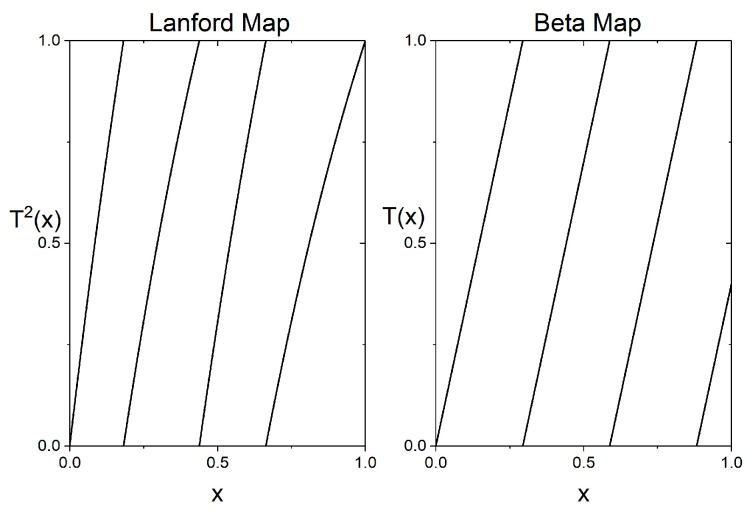
Graphs of the second iterate of the Lanford map (**left panel**) and of the beta map (**right panel**).

We study the time series generated by the second iterate of this map ($T^{2}$), which has full branches and is piecewise expanding. In the left panel of Figure 1,the graph of this map is plotted. As the map is piecewise expanding, the corresponding invariant measure is absolutely continuous. In [2], using a computer-aided proof approach, it was proven that the KS entropy of the second iterate of the Lanford map ($T^{2}$) is contained in the certified interval [1.312,1.318].

The generating partition considered for the symbolisation of the orbits is given by the four maximal subintervals where the map is continuous and it is explicitly given by

$$\left\{\left[0,a_{0}\right),\left[a_{0},a_{1}\right),\left[a_{1},a_{2}\right),\left[a_{2},1\right)\right\},$$

where $a_{0}=\frac{1}{2}\left(5-\sqrt{5+4\sqrt{17}}\right)≃0.182004$, $a_{1}=\frac{1}{2}\left(5-\sqrt{17}\right)≃0.438447$, $a_{2}=\frac{1}{2}\left(5-\sqrt{-3+4\sqrt{17}}\right)≃0.663398$.

### 3.2. Beta Map

We also analyse the time series generated by the beta map T:[0,1]→[0,1] defined by

(24) $$T\left(x\right)=\frac{17}{5}x\;\mathrm{mod}\;1$$

whose graph is plotted in the right panel of Figure 1. This map is piecewise expanding but does not enjoy the Markov property: since ${\left(17/5\right)}^{k}$ is never an integer, there exist dense orbits. For this map the corresponding Lyapunov exponent and hence the value of the KS entropy is given by $L_{\exp}=\ln\left(17/5\right)$.

The generating partition used to symbolise the orbits is the natural one, given by

$$\left\{\left[0,\frac{5}{17}\right),\left[\frac{5}{17},\frac{10}{17}\right),\left[\frac{10}{17},\frac{15}{17}\right),\left[\frac{15}{17},1\right)\right\}.$$

### 3.3. Nonlinear Beta Map

**Figure 2:**
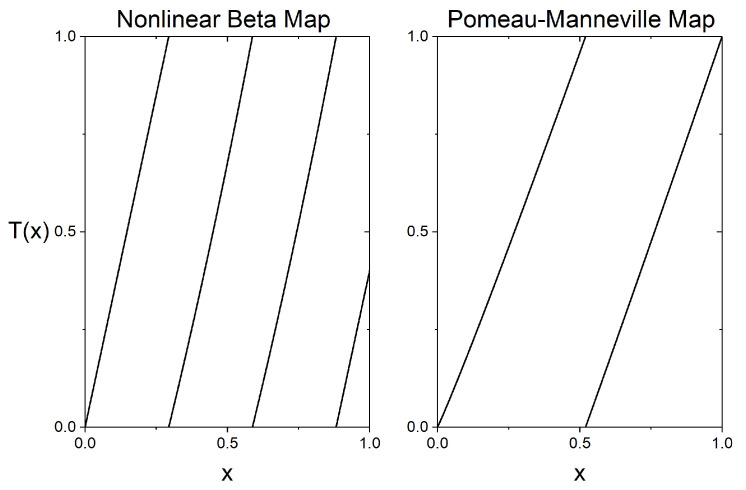
Graphs of the nonlinear version of the beta map (**left panel**) and of the Pomeau–Manneville map for α=1/8 (**right panel**).

In addition to the beta map, we also consider the nonlinear beta map T:[0,1]→[0,1] defined by

(25) $$T\left(x\right)=\left\{\begin{array}{cc} \frac{17}{5}x & 0\leq x\leq \frac{5}{17}\\ \frac{34}{25}{\left(x-\frac{5}{17}\right)}^{2}+3\left(x-\frac{5}{17}\right) & \frac{5}{17}<x\leq \frac{10}{17}\\ \frac{34}{25}{\left(x-\frac{10}{17}\right)}^{2}+3\left(x-\frac{10}{17}\right) & \frac{10}{17}<x\leq \frac{15}{17}\\ \frac{17}{5}\left(x-\frac{15}{17}\right) & \frac{15}{17}<x\leq 1. \end{array}\right.$$

whose graph is plotted in the left panel of Figure 2. This map is really similar to map (24) and includes the absence of the Markov property, but it is nonlinear in the two intervals [5/17,10/17] and [10/17,15/17], where it is defined by two branches of a polynomial of degree two.

The Lyapunov exponent and the KS entropy of this dynamical system were proven in [2] to be in the interval [1.215,1.223].

For the translation into symbolic dynamics of the nonlinear beta map, we take the same natural generating partition as that for the beta map.

### 3.4. Pomeau–Manneville Map

The last dynamical system we consider is an intermittent map of Pomeau–Manneville type. Such maps are classical models of weak chaos and intermittency: they exhibit a neutral fixed point at the origin. The presence of the neutral fixed point is responsible for long laminar phases near 0, which in turn leads to long-range memory effects and to various non-standard statistical properties. The map T:[0,1]→[0,1] is defined by

(26) $$T\left(x\right)=x+x^{1+\alpha }\;\mathrm{mod}\;1,$$

whose graph is plotted in the right panel of Figure 2 for α=1/8. For this value of α, this dynamical system admits a unique absolutely continuous invariant probability measure with an unbounded density, and correlations decay at a polynomial rate. In this non-uniformly expanding setting, the above-mentioned Pesin-type results imply that the Kolmogorov–Sinai entropy $h_{\mu }\left(T\right)$ coincides with the Lyapunov exponent.

In ref. [2] certified bounds for the Lyapunov exponent for the Pomeau–Manneville map (26) were computed, and both the values of the Lyapunov exponent and the KS entropy lie in the certified interval [0.68,0.69].

Analogously to the other three maps, the generating partition for the Pomeau–Manneville map considered for the symbolisation of its orbits is given by the maximal subintervals where the map is continuous. It is given by

$$\left\{\left[0,a_{0}\right),\left[a_{0},1\right)\right\},$$

where $a_{0}≃0.5204$.

In summary, for all four maps, the certified intervals where the KS entropy falls are the following: [1.312,1.318] for the Lanford map, $\ln(\frac{17}{5})≃1.2238$ for the beta map, [1.215,1.223] for the nonlinear beta map, and [0.68,0.69] for the Pomeau–Manneville map. These certified intervals are used as references for the benchmark of the KS entropy estimators in Section 4.

## 4. Results

### 4.1. Description of the Data Analysis

**Figure A1:**
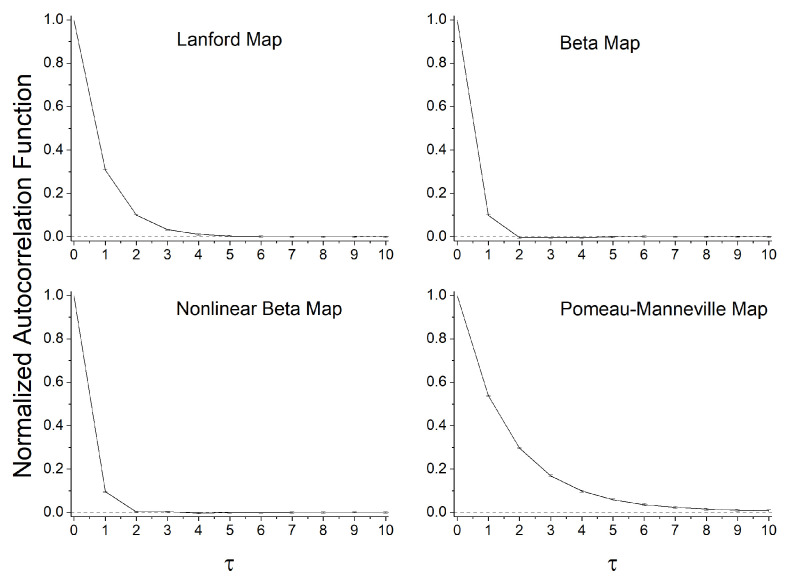
Autocorrelation functions for the time series generated by the four maps. Each panel shows the average autocorrelation function, and the error bars are the corresponding standard deviation values.

For every dynamical system described in Section 3, $N_{r}=200$ orbits were generated using random initial conditions. The i−th ($i=1,2,\ldots ,N_{r}$) orbit consists in a time series ${\left\{x_{l}^{i}\right\}}_{l=1}^{N}$ of *N* data points, and $C_{M}\left(i\right)$ denotes the value of the chosen complexity/entropy estimator estimated using ${\left\{x_{l}^{i}\right\}}_{l=1}^{N}$. The choice of the value of *N* will be discussed in the following. Then, the complexity of the dynamics generated by the chosen map is quantified by the mean value $<C_{M}\left(i\right)>=N_{r}^{-1}\sum_{k=1}^{N_{r}}C_{M}\left(i\right)$ and by the corresponding standard deviation. This is justified by numerical evidence, indicating that the distribution of the values of a given complexity measure, obtained from hundreds of orbits generated with random initial conditions, are approximately Gaussian. As shown in Section 2, the estimation of the entropy/complexity of data series employed in the present work requires the specification of the values of some parameters (such as embedding dimension and lag time). To achieve this aim, it is useful to make some important remarks. As established in the literature, we do not have any a priori well-defined criterium (or general analytical result) that permits us to choose the “best” set of parameter values when estimating the ApEn, SampEn, and PerEn from a time series. Since we are interested in comparing the performance of the above entropy estimators. it is absolutely necessary to avoid any possible bias that could be introduced by the methodological approach adopted choosing such parameter values. Thus, for each entropy estimator, we adopt the same set of parameter values to analyse the time series generated by all four maps used in this work. Let us start with the parameter values required for the application of ApEn and SampEn. As suggested in several research works [8,9,11,48,49,50], for these two entropy estimators. we choose the following parameter values: E=2, r=0.2, and τ=1. We point out that using higher values of *E* for a fixed number of data points of the time series increases the computational cost and reduces the statistical accuracy (lower density of states in the reconstructed phase space). Let us now come to the parameter choice for the estimation of PerEn. First of all, it is important to emphasize that for a given choice of the embedding dimension *E*. it holds true that PerEn ≤log(E+1)/E. In particular, for an uncorrelated signal, we get the equality (more details can be found in Appendix B). For instance, if we choose E=2 we get that the maximum value of PerEn will be ≈0.89588, and therefore it is impossible to estimate higher values of entropy. So, how can we choose the *E* value for all maps? To address this problem we studied the autocorrelation properties of the time series investigated in this work. As shown in Appendix A, the time series generated by the dynamical systems used in this work are characterized by a fast decay of the linear correlation between successive values (see Figure A1). For instance, the normalized autocorrelation function for the case of the Lanford map is about 0.1 for τ=2 (τ being the time lag). This implies that, if we consider the embedding dimension E=4 and τ=1, then the first and the last component of the corresponding embedding vector behave approximately as uncorrelated variables. Therefore, the above remarks lead us to make a sort of compromise between two opposite requirements; thus, we adopt the parameter values E=3 and τ=1 for the estimation of the PerEn for all our time series. In summary, for the estimation of the ApEn and SampEn, we chose the following values of the parameters for all time series: E=2, τ=1 and r=0.2. In contrast, for the estimation of PerEn, we adopted the parameter values E=3, τ=1 for all time series. This methodological approach eliminates any bias arising from an “ad hoc” selection of the parameter values of each method for the different maps. A systematic study on how the parameters influence the values of the estimated ApEn, SampEn and PerEn can be found in Appendix B. A discussion on the computational complexity costs involved in the estimation of ApEn, SampEn, and PerEn can also be found in this appendix.

Let us now discuss the approaches that will be used to select the parameter values for the application of symbolic methods for the estimation of the Kolmogorov–Sinai entropy. To this aim we look for an algorithmic way of determining a good block length *k* to stop at for the Plug-In (PI) method and a good number *k* of pair substitutions to perform for the NSRPS method. We want the two methods to capture all the meaningful dependency properties of the dynamical systems so that on one hand we would tend to take large values of *k*. On the other hand, however, poor statistics effects arise for large values of *k* due to the finiteness of the data, and thus a good trade-off is needed.

For the NSRPS method we define a notion of a significant pair substitution depending on three parameters (a window length *w*, a coefficient δ, and a quantile level *q*), as a substitution that condenses the memory properties significantly more than the next *w* ones, where significance means that the entropy estimate gain due to that particular pair substitution is greater than δ times the *q*-quantile of the *w* subsequent entropy estimate gains. We finally take as the final entropy estimate for the NSRPS method the one corresponding to the last significant pair substitution (see Appendix C.1 for the details). After exploring several values we take w=15, δ=4, q=0.75 and discuss the dependence of the results on the choice of these parameters in Appendix C.1, showing that the results are robust.

Similarly, we define a notion of a significant block length for the Plug-In method, where significance is defined as the entropy estimate gain of block length *k* being greater, in absolute value, to δ times the gain due to block length k+1 (see Appendix C.2 for the details). Our final entropy estimate for the Plug-In method is the one corresponding to the largest significant block length. We take δ=2.5 and discuss the robustness of the results with respect to the choice of δ in Appendix C.2.

In Appendix C we also briefly discuss the algorithmic complexity of the Plug-In and the NSRPS methods.

Before completing this section we consider an important question: what is the dependence of the employed complexity measures on the number of data points (*N*) of the time series? This problem was studied by adopting, for each method, the parameter values described above in this section. The corresponding results for all complexity measures are reported in Table 1. From these data it follows that an acceptable compromise between computational costs and accuracy of the corresponding complexity measures leads us to choose N=100,000 data points.

### 4.2. Comparison of the Performance of the Entropy Estimators

**Figure 3:**
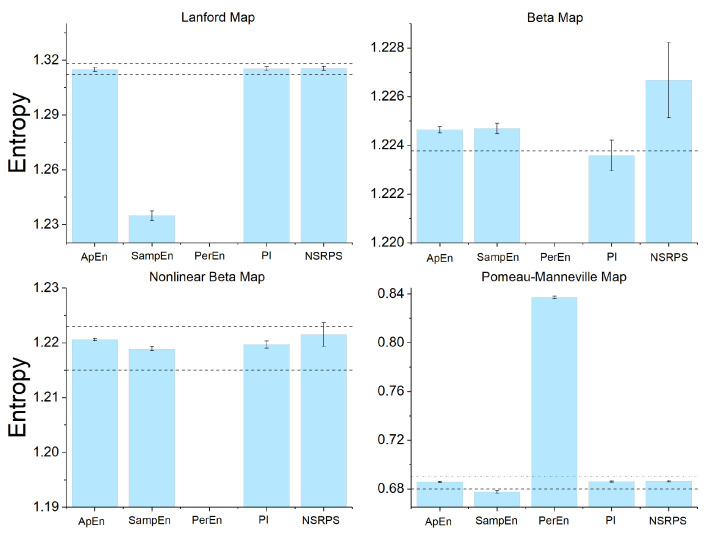
Mean values of the Kolmogorov–Sinai entropy of all maps estimated by the five complexity measures adopted in this work. In all the panels the error bars represent the corresponding values of the standard deviation. The ApEn and SampEn values were obtained using the parameter values E=2, r=0.2, and τ=1, while those of PerEn were obtained using E=3 and τ=1. The PI and NSRPS estimates were obtained with δ=2.5 and with w=15, δ=4, and q=0.75, respectively.

We are now ready to compare the performance of the five entropy measures (ApEn, SampEn, PerEn, Plug-In, and NSRPS) used in this work for estimating the Kolmogorov–Sinai entropy of all four maps. We point out that the corresponding values were estimated, for each method, by using the selected parameter values discussed in the previous section. The mean values of the five entropy estimators, together with the corresponding standard deviations, are plotted in Figure 3. The results for the Lanford map are shown in the top left panel. The inspection of these data shows that the values of ApEn, Plug-In, and NSRPS are all within the exact interval where the value of the Kolmogorov–Sinai entropy of the Lanford map must be. The value of PerEn does not appear in this panel because it is out of the plotted scale (the corresponding mean value can be found in Table 2). Consequently, SampEn and PerEn underestimate the entropy. The top right panel of Figure 3 shows the results for the beta map, for which we know the exact value of the Kolmogorov–Sinai entropy (log(17/5)). It can be seen that only the Plug-In symbolic method produces the best estimation of the value of the entropy for this map. As with the Lanford map, the PerEn measure is out of the range of the plotted entropy values (the corresponding mean value can be found in Table 2). The bottom left panel of Figure 3 shows the results for the nonlinear beta map. In this case the ApEn, SampEn, PI, and NSRPS mean values are all within the certified interval, while the PerEn value is out of the range of the entropy values. Also in this case the PerEn underestimates the entropy value for this map (see Table 2). Lastly, the results for the Pomeau–Manneville map are plotted in the bottom right panel of Figure 3. The inspection of these data shows that the estimators ApEn, Plug-In, and NSRPS produce estimates of the Kolmogorov–Sinai entropy that are all within the certified interval. The SampEn estimator underestimates the KS entropy, while the PerEn mean value overestimates it.

In summary, on the basis of our detailed numerical investigation, we can conclude that for the dynamical systems studied in this work, the more accurate complexity measures are the ApEn, the Plug-In, and the NSRPS symbolic methods. In contrast, the SampEn and the PerEn estimators generally underestimate the entropy of these maps (apart from the case of the Pomeau–Manneville map for which PerEn overestimates the certified range of entropy values). Table 3 provides a schematic summary of the performance of the entropy estimators used in this work.

## 5. Conclusions and Future Directions

In this work we have taken advantage of recent results on computer-assisted, rigorous entropy estimates of certain one-dimensional dynamical systems to build a quantitative benchmark for several widely used entropy estimators for time series. Using four representative maps for which certified estimates for the entropy were available, we compared the output of several entropy empirical estimators against rigorously certified values.

Our numerical experiments show that the behaviour of these estimators depends markedly on the dynamical features of the underlying system. Even in the idealised setting of long, noise-free trajectories, the time-series based estimators PerEn and SampEn tend to exhibit a systematic underestimation of the entropy, particularly pronounced for uniformly expanding maps, and a noticeable sensitivity to the choice of embedding and radius parameters. Among all the complexity measures examined here, the PerEn fails to predict the certified values of entropy for all maps. In contrast, the ApEn and the symbolic estimators (Plug-In and NSRPS with bias correction) provide entropy values that consistently fall within the rigorous error bars for all the four examples, and their variability across realisations remains comparatively small.

Naturally, our study has several limitations. The benchmark is restricted to a certain set of deterministic, one-dimensional systems with absolutely continuous invariant measures for which rigorous computer-aided estimates for the entropy were available in the literature. The chosen examples however span a range of different statistical behaviours, from exponential to polynomial decay of correlations, and include intermittent dynamics. Although these systems are not sufficient to provide a definitive, global ranking of the estimators, our results should be viewed as a first, quantitatively controlled step in this direction. In particular, in line with the previous remarks, we also plan, in a future work, to include in our study the asymmetrical tent map and the critical map. These two maps have been studied using several approaches by different authors and, for well-defined values of the corresponding parameters, they have an absolutely continuous invariant measure and their KS entropies are known exactly [19,51,52,53].

Several extensions are worth pursuing. On the dynamical side, it would be natural to include higher-dimensional systems, more complex invariant measures, and noise. On the data-analysis side, the impact of observation noise and coarse-graining procedures on each estimator should be systematically investigated. Also the use of empirical estimators of the entropy not considered in the present paper and the comparison with the results presented here, as already remarked at the end of the introduction, warrants further work. We believe that the methodology developed here, combining rigorous dynamical input with carefully designed numerical experiments, provides a solid starting point for such future studies and for a more informed use of entropy estimators in applications.

## Figures and Tables

**Table 1:** Dependence of the mean values of the complexity measures on the number of points of the time series. The corresponding standard deviations are reported between parentheses.

Lanford Map	N	ApEn	SampEn	PerEn	Plug-In	NSRPS
	25,000	1.3131 (0.0025)	1.2347 (0.0056)	0.8677 (0.0013)	1.3152 (0.0025)	1.3154 (0.0025)
	50,000	1.3142 (0.0018)	1.2345 (0.0041)	0.8677 (0.0009)	1.3152 (0.0018)	1.3154 (0.0018)
	75,000	1.3146 (0.0014)	1.2347 (0.0033)	0.8677 (0.0008)	1.3153 (0.0014)	1.3155 (0.0015)
	100,000	1.3149 (0.0011)	1.2348 (0.0026)	0.8678 (0.0007)	1.3154 (0.0011)	1.3155 (0.0011)
Beta Map	N	ApEn	SampEn	PerEn	Plug-In	NSRPS
	25,000	1.2235 (0.0004)	1.2247 (0.0007)	0.8803 (0.0011)	1.2239 (0.0011)	1.2309 (0.0039)
	50,000	1.2243 (0.0002)	1.2247 (0.0004)	0.8803 (0.0008)	1.2238 (0.0009)	1.2281 (0.0024)
	75,000	1.2245 (0.0002)	1.2247 (0.0003)	0.8804 (0.0006)	1.2238 (0.0006)	1.2273 (0.0018)
	100,000	1.2246 (0.0001)	1.2247 (0.0002)	0.8804 (0.0005)	1.2236 (0.0006)	1.2267 (0.0015)
Nonlinear Beta Map	N	ApEn	SampEn	PerEn	Plug-In	NSRPS
	25,000	1.2195 (0.0005)	1.2189 (0.0009)	0.8815 (0.0010)	1.2196 (0.0016)	1.2248 (0.0029)
	50,000	1.2202 (0.0003)	1.2189 (0.0006)	0.8816 (0.0007)	1.2197 (0.0008)	1.2237 (0.0024)
	75,000	1.2205 (0.0003)	1.2189 (0.0005)	0.8816 (0.0006)	1.2197 (0.0007)	1.2219 (0.0024)
	100,000	1.2206 (0.0002)	1.2189 (0.0004)	0.8816 (0.0006)	1.2197 (0.0006)	1.2215 (0.0022)
Pomeau–Manneville Map	N	ApEn	SampEn	PerEn	Plug-In	NSRPS
	25,000	0.6855 (0.0009)	0.6774 (0.0022)	0.8373 (0.0019)	0.6861 (0.0009)	0.6867 (0.0008)
	50,000	0.6857 (0.0007)	0.6775 (0.0016)	0.8373 (0.0013)	0.6860 (0.0007)	0.6866 (0.0007)
	75,000	0.6858 (0.0005)	0.6775 (0.0012)	0.8373 (0.0011)	0.6860 (0.0005)	0.6864 (0.0006)
	100,000	0.6858 (0.0004)	0.6775 (0.0011)	0.8373 (0.0010)	0.6859 (0.0005)	0.6863 (0.0006)

**Table 2:** Result summary for the entropy estimation of the maps.

	ApEn	SampEn	PerEn	Plug-In	NSRPS	Certified Value
Lanford	1.3148 (0.0011)	1.2348 (0.0026)	0.8678 (0.0007)	1.3154 (0.0011)	1.3155 (0.0011)	[1.312, 1.318]
Beta	1.2246 (0.0001)	1.2247 (0.0002)	0.8804 (0.0005)	1.2236 (0.0006)	1.2267 (0.0015)	$\ln(\frac{17}{5})≃1.2238$
Nonlinear Beta	1.2206 (0.0002)	1.2189 (0.0004)	0.8816 (0.0006)	1.2197 (0.0006)	1.2215 (0.0022)	[1.215, 1.223]
Pomeau–Manneville	0.6858 (0.0004)	0.6774 (0.0011)	0.8373 (0.0010)	0.6859 (0.0005)	0.6863 (0.0006)	[0.68, 0.69]

**Table 3:** Performance of the adopted complexity measures. The sign + means that the given complexity measure falls within the certified interval where the Kolmogorov–Sinai entropy must be; the sign − means that the corresponding mean value is out.

	ApEn	SampEn	PerEn	Plug-In	NSRPS
Lanford Map	+	−	−	+	+
Beta Map	−	−	−	+	−
Nonlinear Beta Map	+	+	−	+	+
Pomeau–Manneville Map	+	−	−	+	+

**Figure A2:**
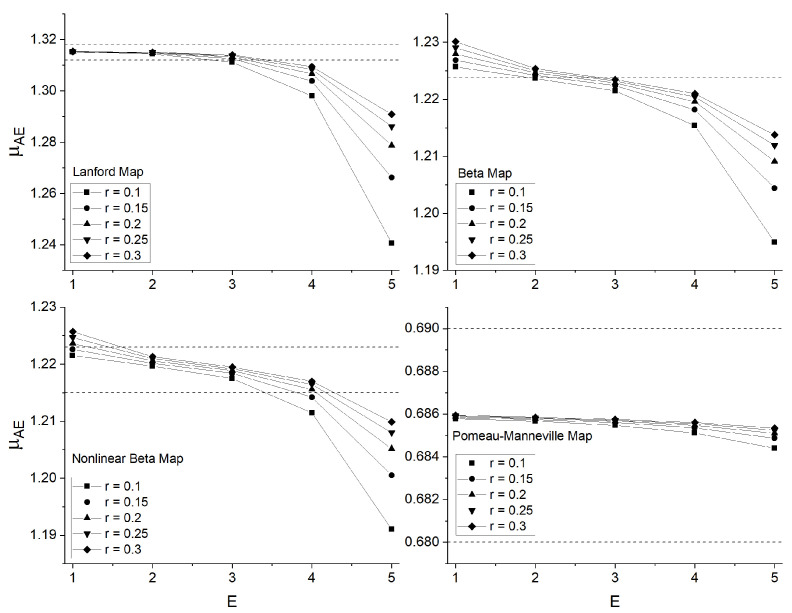
Mean values of ApEn ($\mu_{AE}$) for different values of the embedding dimension *E* and radius *r* of ball in $R^{E}$. Top panels: (**left**) data for the Lanford map; (**right**) data for the beta map. Bottom panels: (**left**) data for the nonlinear beta map; (**right**) data for the Pomeau–Manneville map. For all panels and for each fixed value of *E* and *r*, each data point (or symbol) corresponds to the mean value of ApEn over 200 orbits. The dashed lines define the certified intervals where the Kolmogorov–Sinai entropy (or, equivalently, the Lyapunov exponent) falls.

**Figure A3:**
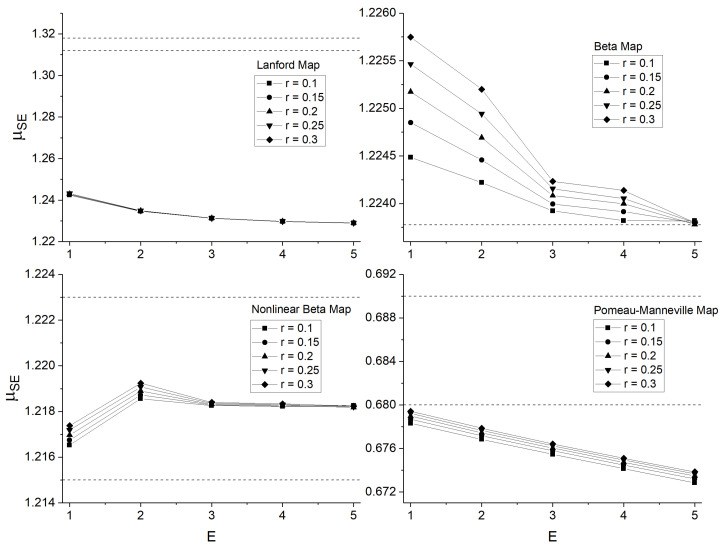
Mean values of SampEn ($\mu_{SE}$) for different values of the embedding dimension *E* and radius *r* of ball in $R^{E}$. Top panels: (**left**) data for the Lanford map; (**right**) data for the beta map. Bottom panels: (**left**) data for the nonlinear beta map; (**right**) data for the Pomeau–Manneville map. For all panels and for each fixed value of *E* and *r*, each data point (or symbol) corresponds to the mean value of SampEn over 200 orbits. The dashed lines define the certified intervals where the Kolmogorov–Sinai entropy (or, equivalently, the Lyapunov exponent) falls.

**Figure A4:**
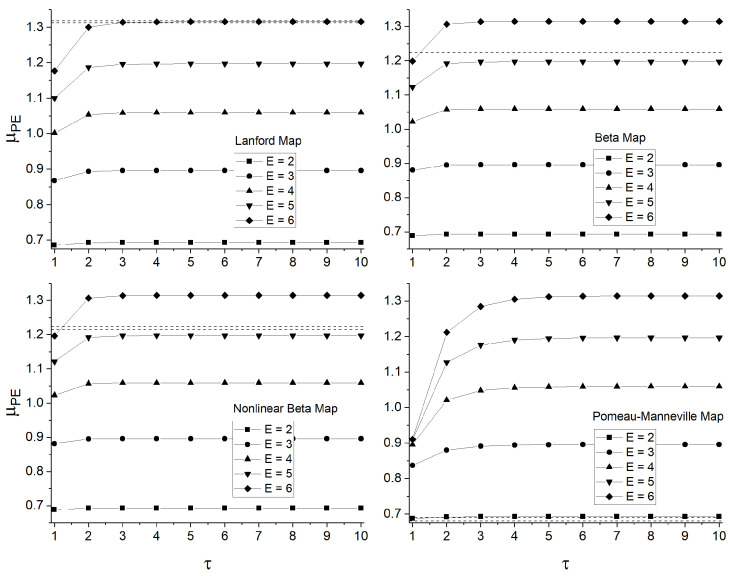
Mean values of PerEn ($\mu_{PE}$) for different values of the embedding dimension *E* and lag time τ. Top panels: (**left**) data for the Lanford map; (**right**) data for the beta map. Bottom panels: (**left**) data for the nonlinear beta map; (**right**) data for the Pomeau–Manneville map. For all panels and for each fixed value of *E* and τ, each data point (or symbol) corresponds to the mean value of PerEn over 200 orbits. The dashed lines define the certified intervals where the Kolmogorov–Sinai entropy (or, equivalently, the Lyapunov exponent) falls.

**Figure A5:**
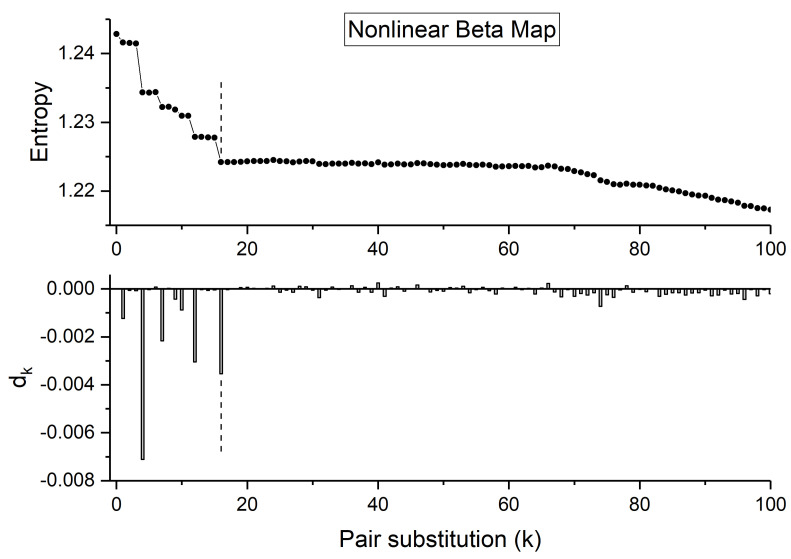
The top graph shows the term $h_{k}^{\mathrm{NSRPS}}=\frac{h_{1}\left(\mu_{k}\right)}{{\bar{Z}}_{k}}$ of Equation (21) as a function of *k*. In the bottom panel the differences $h_{k}^{\mathrm{NSRPS}}-h_{k-1}^{\mathrm{NSRPS}}$ are graphed against the number *k* of pair substitutions. Both graphs refer to the same sample orbit of the nonlinear beta map.

**Figure A6:**
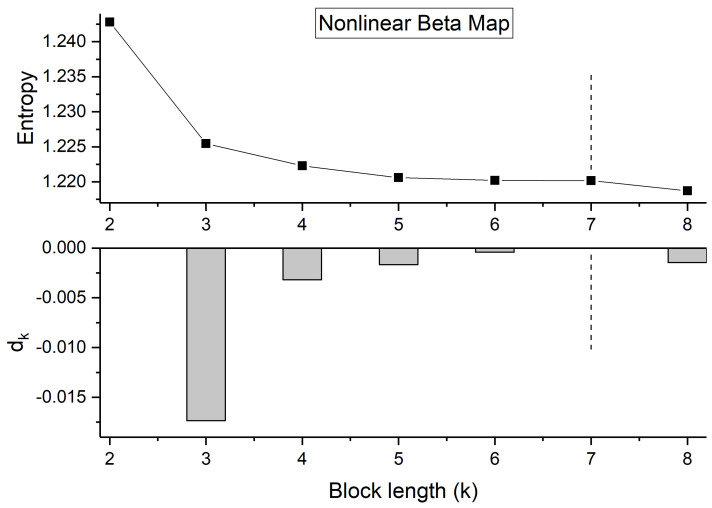
The top graph shows the conditional entropy $h_{k}^{\mathrm{PI}}$ as a function of *k*. In the bottom panel the differences $h_{k}^{\mathrm{PI}}-h_{k-1}^{\mathrm{PI}}$ are graphed against the block length *k*. Both graphs refer to the same sample orbit of the nonlinear beta map.

## Data Availability

The data and the code that support the findings of this study are available from the corresponding author upon request.

## Appendix A. Autocorrelation Properties of the Signals

In this section we discuss the autocorrelation properties of the time series analysed in this paper. For each of them the linear autocorrelation function was computed on windows of $N=10^{5}$ data points. Then, the corresponding autocorrelation functions, obtained using 200 orbits with random initial conditions, were averaged. In Figure A1 the results for all four maps are plotted. The data reported in the top left panel of this figure show that for the time series generated by the Lanford map, the average linear correlation decays quickly to zero (τ≃4). For the beta and the nonlinear beta maps, this decay is faster (τ≃2). In contrast, the autocorrelation function for the Pomeau–Manneville map decays more slowly (τ≃7).

## Appendix B. Dependence of the Values of ApEn, SampEn and PerEn on the Parameter Values

In this appendix we briefly discuss the dependence of the ApEn, SampEn, and PerEn values on the corresponding parameter values of each method. For both ApEn and SampEn the parameters *E* and *r* are varied in the following intervals: E∈{1,2,3,4,5} and r∈{0.1,0.15,0.2,0.25,0.3}, with τ=1. In contrast, for the PerEn, the parameters *E* and τ are varied in the following intervals: E∈{1,2,3,4,5,6} and τ∈{1,2,3,4,5,6,7,8,9,10}. As can be immediately understood, carrying out such simulations for all complexity measures, using serial numerical computation, requires a huge computational time. To be more precise, let us briefly discuss the computational complexity costs associated to the estimation of ApEn, SampEn, and PerEn for a time series of *N* data points. From the definition of ApEn (see Section 2.2), it can be shown that, for a given embedding dimension *E*, the corresponding computational cost scales as $O(EN^{2})$. For small values of *E*, this computational cost can be approximated by $O(N^{2})$. A similar result holds true also for the computational complexity cost of SampEn. Meanwhile, from the definition of PerEn (see Section 2.3), it follows that the corresponding computational cost scales as $O(E\log EN^{2})$. For small *E* values the computational cost can be approximated by O(N). Therefore, on the basis of the previous comments, to drastically reduce these computational costs, Fortran codes with OpenMP parallelization directives were built and run on a multithread machine (64 threads). In the following we explore how the mean values of ApEn, SampEn, and PerEn depend on the corresponding parameter values. Let us start with the ApEn complexity measure. For the case of the Lanford map, the corresponding results are reported in the top left panel of Figure A2. The two dashed lines define the interval where the exact value of the Kolmogorov–Sinai entropy (or Lyapunov exponent) must be contained [2]. For E=1 (or E=2) the corresponding ApEn values are all inside this interval, independently of the values of *r*. Moreover, the increase in the *E* value determines the decrease in the corresponding mean values of ApEn. This can be partially explained by the fact that using the same number of data points in a higher-dimensional reconstructed phase space leads to a decrease in the statistical accuracy (this occurs for all maps). For the beta map the corresponding results (see top right panel of Figure A2) show that for E=2 and r=0.2, ApEn values are close to the exact analytical result (log(17/5)). The increase in the values of the parameter *E* leads qualitatively to the same results found for the Lanford map. The results for the nonlinear beta map are reported in the bottom left panel of Figure A2 and are qualitatively similar to those obtained for the beta map. Lastly, the bottom right panel of Figure A2 shows the result for the Pomeau–Manneville map. In this case for any value of the pair (*E*, *r*), we get entropy values that are within the certified interval. The results for the SampEn are reported in Figure A3. For the Lanford map the corresponding SampEn values are all out the certified interval [1.312,1.318]. Similar conclusions also hold for the Pomeau–Manneville map (bottom right panel). For the beta map the corresponding results are reported in the top right panel of Figure A3, and they show that for any value of the pair (*E*, *r*), the values of SampEn are close to the exact analytical value of the Kolmogorov–Sinai entropy (for E=1, for the worst case the ratio $\left|\right|\mu_{SE}\left(r=0.3\right)-\log\left(17/5\right)\left|\right|/\log\left(17/5\right)$ is of the order of 0.18%). Meanwhile, for the nonlinear beta map, all values of SampEn are within the certified interval (see the bottom left panel of Figure A3). Lastly, let us discuss the results for PerEn obtained by varying the parameter *E* and τ. The corresponding findings, for all four maps, are reported in Figure A4. The inspection of these data and a comparison with those already seen for the ApEn and SampEn show that the PerEn values exhibit a strong dependence on the embedding dimension. For instance, for all the maps, the values of PerEn corresponding to (E,τ)=(2,3) and (E,τ)=(6,3) differ approximately by 0.6. Moreover, for each map, this dependence on *E* is in the same direction; i.e., the increase of *E* leads to the increase in the values of PerEn. In addition, the inspection of these data shows that the values of PerEn plateau approximately for τ≥3. This behaviour of PerEn has a simple explanation: for sufficiently large τ values, the components of the embedding vectors are uncorrelated (see the results for the autocorrelation functions of the analysed time series in Figure A1). Then, in such a situation, there is a convergence of the PerEn values towards the limiting value ln(E+1)!/E (corresponding to the case of a purely random time series).

## Appendix C. Details on Determining the Entropy Rate Estimates with Symbolic Methods

Working with finite samples means of course that one must stop at a certain order *k* when estimating the entropy rate by Equation (20) or at a certain number *k* of pair substitutions when using Equation (21). As already pointed out, such orders should be large enough to capture the dependency properties of the system. In fact, they could be as small as 1 for systems giving rise to independent symbolic dynamics and should be much larger for long memory systems. The question arises as to how good values can be chosen for them.

In [17] the choice for the order *k* in the Plug-In estimator was made as the largest integer $\bar{k}<\log_{m}\left(N\right)$, with *m* being the cardinality of the generating partition and *N* the sample length. The number *k* of pair substitutions to perform in the NSRPS method was instead determined by the frequency of the most frequent symbol pair: the pair substitution procedure stopped as soon as such frequency went below an arbitrary threshold. Such choices performed reasonably well, but their greatest shortcoming is that they do not depend on the memory properties of the system. A better, more system-specific means to determining them is desirable.

Before going deeper into this, we briefly note that the algorithmic complexity of the Plug-In and the NSRPS method, as a function of the sample length *N*, is O(Nlog(N)). In fact, at each step *k*, measuring the empirical frequencies of the finite blocks necessary to calculate the entropies $H_{k}$ in Equations (20) and (21), plus performing a pair substitution in the NSRPS method, requires parsing the whole symbolic sequence a constant number of times, thus resulting in a factor *N*. Then, as we implemented the two methods, a total number of steps (the block lengths in the Plug-In method and the pair substitutions in the NSRPS method) proportional to log(N) was performed.

### Appendix C.1. NSRPS

Figure A5 shows the dependence of the NSRPS method estimate on the number *k* of pair substitutions for the nonlinear beta map. It can be clearly seen that some pair substitutions lead to a significant decrease in the entropy estimate, while others leave the estimate more or less unchanged, meaning that the former substitutions condense the memory properties of the symbolic series more than do the latter. After all the significant dependencies were condensed by the sequence of pair substitutions, a plateau in the entropy estimate was observed, followed by a steady decrease due to the finiteness of the symbolic sequence and its progressive shortening leading to scarce statistics. A good number of pair substitutions to perform lies after all the significant ones, in order to capture all the meaningful dependency properties, and before the spurious decrease in the estimate due to the poor statistics effects.

In order to implement an algorithmic choice respecting these considerations, we first define what a significant substitution is. Let $h_{k}^{\mathrm{NSRPS}}=\frac{h_{1}\left(\mu_{k}\right)}{{\bar{Z}}_{k}}$ be the entropy estimate after *k* pair substitutions and let $d_{k}=h_{k}^{\mathrm{NSRPS}}-h_{k-1}^{\mathrm{NSRPS}}$ be the difference sequence. Let *w* be a window length, δ>0 a coefficient, and 0<q<1 a quantile level. We say that the pair substitution *k* is significant if

(A1) $$-d_{k}>\delta \cdot Q\left(\left\{d_{k+1},\ldots ,d_{k+w+1}\right\},q\right),$$

where Q(A,q) is the *q*-quantile of set *A*. We finally choose $\bar{k}$ as the last significant substitution, that is, the largest *k* satisfying (A1). The entropy estimate will then be $h_{\bar{k}}^{\mathrm{NSRPS}}$.

After exploring several combinations of values for the involved parameters and their impact on the entropy estimate, we finally fix w=15, δ=4, q=0.75. The entropy estimates are quite robust with respect to the specification of these parameters around the chosen values. In Table A1, we collect the average entropy estimates corresponding to various specifications of the parameters for the four maps.

**Table A1:** Average entropy estimates obtained with the NSRPS method corresponding to the specified values of the parameters *w*, δ, and *q* in the definition of a significant pair substitution (A1). The last row (w=15, δ=4, q=0.75) corresponds to the chosen parameter values and to our final estimates.

	Lanford	Beta	Nonlinear Beta	Pomeau–Manneville
w=15, δ=3, q=0.7	1.3152	1.2255	1.2191	0.6859
w=15, δ=5, q=0.8	1.3155	1.2276	1.2224	0.6866
w=12, δ=4, q=0.7	1.3154	1.2264	1.2209	0.6862
w=18, δ=4, q=0.8	1.3155	1.2271	1.2217	0.6864
w=12, δ=3, q=0.75	1.3153	1.2257	1.2194	0.6859
w=18, δ=5, q=0.75	1.3155	1.2274	1.2223	0.6865
w=15, δ=4, q=0.75	1.3155	1.2267	1.2215	0.6863

### Appendix C.2. Plug-In

Figure A6 shows the dependence of the Plug-In method estimate on the block length *k* for the nonlinear beta map.

The typical behaviour of the entropy estimates shows an initial significant decrease (here for block lengths k=2,…,5) due to the sequence dependency properties being taken into account by the growing block length. Then a plateau follows of essentially equal values (here from k=5 to k=7), where larger block lengths do not capture significantly more dependency properties than do the shorter ones. Finally, at the largest block lengths, a non-meaningful decrease happens due to the insufficient statistics of high block lengths.

Of course, we want to select a good entropy estimate after all the significant memory properties have been captured by a sufficiently large block length but before any spurious decrease due to scarce statistics.

In a similar fashion to what we did for the NSRPS method, we first define a significant block length. Let $h_{k}^{\mathrm{PI}}$ be the entropy estimate for block length *k* and let $d_{k}=h_{k}^{\mathrm{PI}}-h_{k-1}^{\mathrm{PI}}$ be the difference sequence. Let δ>0 be a coefficient. We say that the block length *k* is significant if $d_{k}<0$ and

(A2) $$d_{k}>\delta d_{k+1}.$$

We finally choose $\bar{k}$ as the largest significant block length and the entropy estimate will be $h_{\bar{k}}^{\mathrm{PI}}$.

We take δ=2.5 after exploring several values. In Table A2 we report the average entropy estimates of the four maps corresponding to δ=2,2.5,3. The results show that the entropy estimates are very robust with respect to the choice of this parameter’s value for all the four maps. We conclude by remarking that similar results were obtained for the selection of the parameters values for the other maps.

**Table A2:** Average entropy estimates obtained with the Plug-In method corresponding to δ=2, 2.5 (chosen value), and 3 in (A2).

	Lanford	Beta	Nonlinear Beta	Pomeau–Manneville
δ=2	1.3154	1.2237	1.2197	0.6859
δ=2.5	1.3154	1.2236	1.2197	0.6859
δ=3	1.3154	1.2236	1.2196	0.6859
